# Socio-demographic determinants of obesity indexes in Iran: findings from a nationwide STEPS survey

**DOI:** 10.15171/hpp.2018.25

**Published:** 2018-07-07

**Authors:** Ali-Asghar Kolahi, Alireza Moghisi, Yalda Soleiman Ekhtiari

**Affiliations:** ^1^Social Determinants of Health Research Center, Shahid Beheshti University of Medical Sciences, Tehran, Iran; ^2^Deputy General Director for NCDs, National Focal Person for STEPS Survey & Director for Injury Prevention, Ministry of Health and Medical Education, Tehran, Iran

**Keywords:** Body mass index, Obesity, Abdominal obesity, Hypertension, Type 2 diabetes mellitus, Cardiovascular diseases

## Abstract

**Background:** Overweight and obesity are considered as non-communicable diseases (NCDs) risk factors with increasing prevalence worldwide. This nationwide study aimed to determine the socio-demographic determinants of obesity indexes in Iran.

**Methods:** This cross-sectional study was conducted based on data from the sixth round of nationwide Stepwise approach to surveillance (STEPS) survey in the 31 provinces of Iran in 2011. A total of 9878 people aged ≥20 years were selected using a multi-stage cluster sampling method. Data were collected through three steps questionnaire including ecological, physical and biochemical measurements. We assessed body mass index (BMI), waist circumference(WC) and waist-hip ratio (WHR) as the main indicators of obesity.

** Results:** In this study, the weighted prevalence of overweight and obesity were 34.5% (95% CI:33.6, 35.5) and 21.5% (95% CI: 20.7, 22.3), respectively. The mean ± standard deviation (SD)of BMI among men and women were 25.4±4.4 and 26.9±5.2 kg/m^2^ respectively. Older age,living alone, having lower education level, being housewife or retired were associated with overweight and obesity (P<0.05). Urbanization was positively associated with BMI and WC.Being obese or overweight was associated with having a history of type 2 diabetes mellitus (T2DM), hypertension (HTN) and cardiovascular diseases (CVDs).

**Conclusion: ** The prevalence of overweight in this study was less than global rate while it was vice versa for obesity prevalence. Some socio-demographic characteristics were identified as determinants of obesity which should be considered for planning preventive and control action plans.

## Introduction

Overweight and obesity are the major risk factors for non-communicable diseases (NCDs) such as type 2 diabetes mellitus (T2DM), elevated blood pressure (BP), cardiovascular diseases (CVDs), some cancers and musculoskeletal disorders.^1^ According to World Health Organization’s (WHO’s) report, 1.9 billion of adults ≥18 years were overweight and over 600 million were obese in 2014^2^ and by the year 2030, 2.16 billion people are expected to be overweight and 1.12 billion obese.^3^ Along with other countries in the world, obesity is considered as one of the most important public health threats in Asian countries, including Iran.^4^‏ Over an 8-year period (1999-2007), the prevalence of overweight and obesity among Iranian adults has increased significantly.^5^ Iran is one of the developing countries that has experienced social and economic transition, cultural changes and urbanization conditions^6^ which can affect the prevalence of NCDs and their risk factors including overweight and obesity.^7^ The main indicators of obesity are body mass index (BMI), abdominal obesity which is assessed using the waist circumference (WC) and waist-hip ratio (WHR).^3^ Studies showed these indicators are associated with NCDs morbidity and mortality such as CVDS and T2DM.^8-10^ Thus, one the aims of Global Action Plan for the Prevention and Control of Non-Communicable Diseases 2013-2020 by WHO is to stop obesity by 2025.^11^

Epidemiological studies showed that socio-demographic characteristics and namely lifestyles of individuals in any region are among the most important determinants of overweight and obesity.^3^ Therefore, determining the distribution of socio-demographic determinants of overweight and obesity can be helpful in designing actions plans to promote a healthy lifestyle and prevention and control of overweight and obesity. The WHO STEPwise approach to surveillance (STEPS) for NCDs risk factors is a WHO’s recommended approach to determine public health priorities as a baseline for healthy interventions through determining prevalence of risk factors for NCDs such as biological and modifiable behavioral risk factors by standardized STEPS instrument for standard data collection.^12^ This nationwide study aimed to determine socio-demographic determinants of obesity indexes as the risk factors for NCDs in Iran.

## Materials and Methods

### Design and population

This cross-sectional study was conducted based on data from the sixth round of population-based nationwide STEPS survey on NCDs risk factors in Iran in 2011 under the supervision of WHO and with the fiancial support of the Iranian Ministry of Health and Medical Education.^13^ All Iranian people aged 6 to 70 years who lived in the 31 provinces of Iran were considered as the target population of this nationawide survey. Of course, in the present study we only assessed the characteristics of participants aged ≥20 years. In nationwide survey (main survey), 12 000 people were selected as a sample size (considering the goal of survey) through multi-stage cluster random sampling method. The minimum number of samples in each province proportionate to the population of the province is selected. Sampling procedures in this study were:

- Determining primary sampling units (PSUs): Each PSU consisted of a big city or a combination of several small cities.
- Selecting a systematic random sample from PSU list.
- Considering urban/rural as secondary sampling unit (SSU) and eventually selecting 12 SSUs based on a systematic random method from each PSU.
- Determining executive clusters including 20 households in each SSU (20 samples in each executive cluster)
- Selecting random households by postal code and randomly selecting eligible persons from each selected household by KISH method. In KISH method, first researchers provides a list of people aged 6 to 70 years per household. At each age group, men and women are sorted from the oldest to the youngest. Then, in the standard table of the KISH, they found a house with the head of the column equal to the last digit on the right side of the household code and whose head of the row was equal to the total number of eligible people of households from that age group. A person ranked equal to the number inside this house was enrolled in the study.

### Implementation and data collection

Based on STEPS approach, in the main survey data collection consisted of three steps:

- Collecting demographic information and behavioral measurements: through interview with selected participants.
- Measuring antropometric feautures including measuring height, weight, waist circumference, hip circumference and BP.
- Biochemical investigations including measuring blood sugar, lipid and A1C hemoglobin, which is not discussed in this cross-sectional study.

The trained interviewers and executive teams carried out all measurements. The city and province observers controlled and monitored periodically these measurements and reported to higher level officials.

At beginning of visiting to each household, trained interviewers explained the goals of the study and after obtaining verbal informed consents from eligible participants, they completed the questionnaires through face to face interview technique and measured physical and biochemical measurments on that day or on another day at participants’ convenience. The BMI ranges considered in this study were underweight: under 18.5 kg/m^2^, normal weight: 18.5 to 24.9, overweight: 25 to 29.9, obese: 30 and over 30. In addition, according to the definition of WHO abdominal obesity is defined as a WHR above 0.90 for males and above 0.85 for females. A WC of 102 centimetres or more in men and 88 centimetres or more in women are considered as abominal obesity.

### Questionnaire

The questionnaire of the main survey included 103 questions and 2 main parts (General, demographic information questions and the risk factors for NCDs questions). The focus of this cross-sectional study was on the socio-demographic determinants of overweight and obesity. In this regard, we assessed the related questions from the main questionnaire including:

- General information questions: Demographic information including questions about age, gender, residency, level of education, occupation status, ethnicity and family size
- Question about a history of high BP in the person (Yes/No)
- Question about a history of T2DM in the person (Yes/No)
- Question about history of CVDs in the person (Yes/No)
- Physical measurements including BP, weight, height and WC and calculating WHR and BMI.

### Statistical analysis

All analyses were performed using Epi Info version 6 software (CDC, Atlanta, USA) at first and then SPSS version 21 (IBM Corp., Chicago, IL, USA). We calculated the prevalences for total population using weighting based on gender. We used the mean ± Standard deviation (SD) and frequency (%) as the descriptive methods and chi-square, ANOVA and independent *t* tests as the analytical tests for data analysis. *P* < 0.05 was considered statistically significant.

## Results

### Socio-demographic characteristics of the study population

In the main survey, of the 12 000 people, 1809 were excluded because of not being at home after two visits and not willingness to participate in the study (response rate = 84.9%). The data of 313 people were deleted due to incomplete completion of the questionnaire.

In this study we assessed the data of 9878 people aged ≥20 years (41% male and 59% female). We classified people into 5 age groups (20-29, 30-39, 40-49, 50-59 and ≤60 years old). The age group of 20-29 years old had the highest percentage of participants (27.9%). 69.9% of participants were in urban areas while 30.1% were in rural areas. The family size of most participants (67.3%) were 2. In terms of education level and occupation status, the highest percentage of education level was related to primary/secondary/tertiary level of education (37%) and 48.2% of them were housefives. The ethnicity of half of participants was Fars.

### Socio-demographic characteristics associated with BMI 

The mean ± SD of BMI in this study were 25.4±4.4 in men and 26.9 ± 5.2 kg/m^2^in women. 36.2% (95% CI: 35.3, 37.2) of participants had normal BMI. The weighted prevalence of overweight and obesity were 34.5% (95% CI: 33.6, 35.5) and 21.5% (95% CI: 20.7, 22.3), respectively based on BMI. In terms of gender, 36.2% (95% CI: 34.8, 37.7) of men and 33.3% (95% CI: 32.1, 34.5) of women were overweight and 13.9% (95% CI: 12.8, 15) of men and 26.7% (95% CI: 25.6, 27.9) of women were obese. Except for the 20-29 years old age group, most participants in other age groups were overweight and obese and most rate of normal BMI were oberved among participants aged 20-29 years (53.9%). The mean ± SD of BMI among participants in 40-49 years old age group was significantly higher than others (F = 224.79, *P *< 0.001).The majority of residents in urban areas were overweight/obese (61.4%) and the mean ± SD of BMI among residents in urban areas was significantly higher those in rural areas (t = 10.2,* P *< 0.001). In terms of gender, the majority of men and female were overweigh/obese (50.1% (95% CI: 48.6, 51.7) and 60.1% (95% CI:58.8, 61.2), respectively) however, this rate among women was higher than men (*P*<0.001). The mean ± SD of BMI in female was significantly higher than men (t = -15.6, *P *< 0.001). Except for the Baloch and Sistani ethnicities, most people in other ethnicities were obese and overweight. The mean ± SD of BMI among participants of Gilak ethnicity was significantly higher than others (F = 14.09, *P *< 0.001). In terms of education level and occupation status, lower rate of overweight/obesity was only seen among people with university degree (48.3%). The mean ± SD of BMI among illiterate people was signifcantly higher than others (F = 76.77, *P *< 0.001). The highest rates of overweight/obesity were observed among retired people (68.8%). Overweight/obesity status based on BMI according to socio-demographic characteristics of the participants are presented in Table 1.

### Socio-demographic characteristics associated with abdominal obesity related to WC 

As shown in Table 2, the majority of participants aged 50-59 and ≤60 years old had abdominal obesity (51.2% and 54.7%, respectively). The majority of urban and rural residents had no abdominal obesity (60% and 62.8%, respectively). Dramatically, abdominal obesity was obseved among 54.3% of female while only 17.4% of men had abdominal obesity. The highest rate of abdominal obesity (43.7%) was observed among Turkoman ethnicity. Most of illiterate people (56.4%), alone people (54.3%) and housewives (58.5%) had significantly higher WC than others.

### Socio-demographic characteristics associated with WHR

The highest rate of WHR (75.6%) was observed among participants aged ≤60 years. The majority of female and men had WHR above normal (*P* = 0.01) and this ratio was slightly higher in women than men (55.2% vs. 52.6%). The Turkoman ethnicity had still higher ratio than others (66.4%). Like WC, WHR was higher among illiterate (71.3%) and alone people (66.5%) than others. However. in terms of occupation status, retired participants had the highest WHR than others (71%). WHR status according to socio-demographic characteristics of participants are presented in Table 3.

### The status of elevated BP, CVDs and T2DM according to BMI, WC and WHR 

As shown in Table 4, having a history of elevated BP was significantly higher in overweight people followed by obese people than others (39.1% and 38.6%, respectively). This history was also higher among participants with WC and WHR above normal (65.2% and 74.3%, respectively). Having a history of CVDs was higher among overweight people and followed by obese people (38.6% and 32.7%, respectively). WC and WHR above normal were observed among people with CVDs compared to people without CVDs (56.9% and 72.6%, respectively). On the other hand, among participants with WC and WHR above normal, having a history of T2DM was observed more than others (66.2% and 79.3%, respectively). T2DM was found among 41.2% of overweight and 38.3% of obese participants.

### The status of the mean of systolic and diastolic BP according to BMI, WC and WHR

Results of independent *t* test showed that the mean±SD of systolic BP among participants with WC above normal was significantly higher than others (130.4±20.6 vs. 121.5±17.9 mm Hg) (t = 21.1, *P *< 0.001, 95% CI: 8-9.7). The mean ± SD of diastolic BP was also higher among participants with WC above normal than others (81.9±12 vs. 76.7±11 mm Hg) (t = 20.4, *P *< 0.001, 95% CI: 4.6-5.6).

In terms of WHR, results showed that the mean ± SD of systolic BP was significantly higher in participants with WHR above normal compared to people with normal this ratio (129.3±20.3 vs. 119.7±17.1 mm Hg) (t = 24.2, *P *< 0.001, 95% CI: 8.8-10.3). On the other hand, the mean±SD of diastolic BP also had the same status and among participants with WHR above normal was significantly highe than others (80.7±12 vs. 76.4±11.2 mm Hg) (t = 17.7, *P *< 0.001, 95% CI: 3.8-4.8).

Results of one-Way-ANOVA test showed that the highest mean±SD of systolic BP was belongs to obese participants (131.6±20.5 mm Hg, F = 218.8, *P *< 0.001). The highest mean±SD of diastolic BP was also belongs to obese participants (82.9±11.9 mm Hg, F = 256.5, *P *< 0.001).

## Discussion

According to WHO’s report, 39% of adults ≥18 years old were overweight and 13% were obese in 2014.^2^ Considering our findings, the prevalence of overweight in our study was lower than global rate while it was vice versa for obesity prevalence.

In the first nationwide survey of overweight and obesity prevalence among Iranian adults (2004-2005), the age-adjusted means for BMI was 24.6 in men and 26.5 kg/m^2^ in women.^7^ Considering the results of this study, the mean of BMI in both sexes slightly increased in this time period. In addition, according to the Global Health Observatory (GHO) data by WHO, the mean BMI (age-standardized estimate) among over 18 male and female Iranians in 2014 were 25.3 [24.6-25.9] and 27.0 [26.4-27.7], respectively. The results of our study is very close to this report.^14^

In addition, our data showed that several socio-demographic characteristics were associated positively or negatively with overweight and obesity. Similar to the results of most studies^15-17^ overweight was more common among men while the reverse was the case for prevalence of obesity and the rate of obesity was higher in women than men. In our study abdominal obesity was more prevalent in women than men though in most similar studies this rate was higher in men than women.^6,7,9,18^ However, each region of any country has a different pattern of prevalence for overweight and obesity due to sociocultural issues differences. In the most studies in Iran, physical inactivity has been identified as the main cause of higher prevalence of obesity in women than men.^4,6^ On the other hand, it should be noted that obesity prevention particularly in women is more important because overweight and obesity can expose women to gestational problems and birth complications.^19^

In terms of age group, participants aged ≤60 years had BMI, WC and WHR above normal in our study. Of course, in different studies, these rates among different age groups were more prevalent but the positive realtionship between these rates with older age has been confirmed in all of them.^3,4,7,14,15,20,21^ Our findings showed that the level of education is a essential determinant for overweight and obesity so that in our study overweight/obsity was more prevalent in people with lower level of education. A negative association of overweight and obesity with level of education attained has been proven in other similar studies.^3,4,14,20,21^ In a study on country–level data related to overweight prevalence in 192 countries from 2002, 2005, and 2010, higher education level was posotively associated with overweight.^22^

In terms of occupation status, overweight/obesity based on BMI and WHR above normal were higher among retired people than others and WC above normal was higher among housvife women than others. The higher prevalence rates in these two occupational groups are due to lower level of physical activity among these people than other occupations. Obesity indexes were also higher among alone people compared to others. These people were mostly the elderly and had low physical activity. In our study residence in urban areas was also a determinant for overweight and obesity. This finding that urbanization is a one of the main resons for obesity has been confimed in other similar studies in Iran and other countries.^6,7,17,19,22,23^ Higher prevalence of overweight/obesity among some ethnicity in this study is needed further studies to explore the causality of this association.

According to the our findings, among overweight and followed by obese participants and also participants with WC and WHR above normal, the history of elevated BP, CVDs and T2DM were significantly observed more than others. The mean of systolic and diastolic BP were higher among obese participants and those with WC and WHR sbove normal.

Since overweight and obesity are well-established as main risk factors for T2DM, elevated BP and following that CVDs,^4,8,24-26^ the above mentioned findings highlight the necessity of planning and implementation of obesity prevention programs in our country.

### Strengths and limitations of the study

This study is important because draws a picture of socio-demographic distribution of overweight and obesity patterns based on data from a nationwide population-based survey representative of 20 years old and above Iranians. Providing such a picture can identify the specific socio-demographic characteristics of the target population that affect the prevalence of overweight and obesity. Implementation nationwide preventive and control actions using the preventive appropraite strategies to high risk population groups require such socio-demographic pattern.

Notably, the main survey was a population-based study which can lead to underestimating these prevalence rates. On the other hand, further studies are needed to explore causality of the relationship between specific socio-demographic characteristics of target population with the risk factors for overweight and obesity and interaction between lifestyle and obesity.

## Conclusion

Our study showed the growing prevalence of overweight and obesity as compared with previous studies in Iran, as well as other countries in the world. However, the prevalence of overweight in this study was less than global rate while it was vice versa for obesity prevalence. Socio-demographic conditions were identified as determinant factors of overweight and obesity. Some of these factors significantly influenced the prevalence of overweight and obesity in our study, including being female, older age, less than education level (especially illiteracy), being retired and housevife (due to physical inactivity), being alone and urbanization. Being obese or overweight was associated with having the history of T2DM, hypertension (HTN) and CVDs. Identifyong these factors can help health policy makers of our country to develop and implement preventive and control action plan considering these factors.

## Ethical approval

The study protocol conformed to the ethical guidelines of the 1975 Declaration of Helsinki, and the Ethics Committee of Shahid Beheshti University of Medical Sciences under the ethics code IR.SBMU.RETECH.REC.1394.121 approved it. Researches considered all the principles of medical ethics, including the consent of the participants, the confidentiality of personal data and free measurements.

## Competing interests

The authors declare that they have no competing interests.

## Authors’ contributions

All authors contributed to this work, including study concept and design, analysis and interpretation of data, drafting of the manuscript, critical revision of the manuscript for important intellectual content, statistical analysis, administrative, technical, and material support and study supervision. All authors read and approved the final manuscript.

## Acknowledgments

This study has been funded and supported by Shahid Beheshti University of Medical Sciences. The study was a medical student’s thesis.

**Table 1:** Overweight/obesity status based on BMI according to socio-demographic characteristics in the study population

**Characteristics**		**Overweight/ Obesity** ^a^		***P *** **value** ^b^
		**Yes**	**No**	
		**No. (%)**	**No. (%)**	
Age group (y)	20-29	1013 (38.1)	1643 (61.9)	<0.001
	30-39	1004 (57.9)	731 (42.1)	
	40-49	950 (69.1)	424 (30.9)	
	50-59	1429 (69.6)	624 (30.4)	
	≤60	1135 (67.6)	545 (32.4)	
Residency	Urban	4084 (61.4)	2563 (38.6)	<0.001
	Rural	1447 (50.8)	1404 (49.2)	
Sex	Male	2028 (52.3)	1851 (47.7)	<0.001
	Female	3502 (62.3)	2116 (37.7)	
Ethnicity	Baloch	92 (36.8)	158 (63.2)	<0.001
	Turk	1270 (60.2)	840 (39.8)	
	Turkoman	79 (59.40	54 (40.6)	
	Sistani	45 (48.9)	47 (51.1)	
	Arab	119 (56.1)	93 (43.9)	
	Fars	2764 (58.4)	1965 (41.6)	
	Kurd	371 (52.3)	339 (47.7)	
	Gilak	250 (63.8)	142 (36.2)	
	Lor	476 (63)	279 (37)	
	Multiethnicity	23 (51.1)	22 (48.9)	
Education level	No education	1583 (64.1)	885 (35.9)	<0.001
	Primary/secondary/tertiary degree	2168 (61.7)	1346 (38.3)	
	High school degree	1100 (52.3)	1005 (47.7)	
	University degree	679 (48.3)	728 (51.7)	
Family size	1	213 (66.6)	107 (33.4)	<0.001
	2-4	3767 (59)	2622 (41)	
	5 and above	1536 (55.5)	1234 (44.5)	
Occupation status	Housewife	3019 (66.7)	1509 (33.3)	<0.001
	Retried	478 (68.8)	217 (31.2)	
	Unemploed	226 (42.2)	309 (57.8)	
	Worker and employee	608 (49.9)	611 (50.1)	
	Self-employed	956 (52)	882 (48)	
	Student and soldier and unpaid work	191 (31.9)	408 (68.1)	

Abbreviation: BMI, body mass index. ^a^ BMI ranges: underweight: under 18.5 kg/m2, normal weight: 18.5 to 24.9, overweight: 25 to 29.9, obese: 30 and over 30. ^b^ *P* values are from chi-square test.

**Table 2:** Abdominal obesity related to WC according to socio-demographic characteristics in the atudy population

**Characteristics**		**Abdominal obesity**		***P *** **value** ^a^
		**Yes**	**No**	
		**No. (%)**	**No. (%)**	
Age group (y)	20-29	450 (17.7)	2093 (82.3)	<0.001
	30-39	595 (34.6)	1124 (65.4)	
	40-49	658 (48)	712 (52)	
	50-59	1034 (51.2)	985 (48.8)	
	≤60	899 (54.7)	744 (45.3)	
Residency	Urban	2598 (40)	3904 (60)	0.01
	Rural	1038 (37.2)	1754 (62.8)	
Sex	Male	664 (17.4)	3152 (82.6)	<0.001
	Female	2972 (54.3)	2506 (45.7)	
Ethnicity	Baloch	63 (26.3)	177 (73.8)	<0.004
	Turk	808 (40)	1212 (60)	
	Turkoman	59 (43.7)	76 (56.3)	
	Sistani	29 (35.4)	53 (64.6)	
	Arab	89 (41.4)	126 (58.6)	
	Fars	1789 (38.4)	2870 (61.6)	
	Kurd	281 (40.1)	419 (59.9)	
	Gilak	161 (41.7)	225 (58.3)	
	Lor	311 (42)	430 (58)	
	Multiethnicity	17 (37)	29 (63)	
Education level	No education	1371 (56.4)	1061 (43.6)	<0.001
	Primary/secondary/tertiary degree	1369 (39.7)	2080 (60.3)	
	High school degree	583 (28.5)	1460 (71.5)	
	University degree	313 (22.9)	1054 (77.1)	
Family size	1	172 (54.3)	145 (45.7)	<0.001
	2-4	2460 (39.4)	3779 (60.6)	
	5 and above	994 (36.6)	1725 (63.4)	
Occupation status	Housewife	2612 (58.5)	1851 (41.5)	<0.001
	Retried	225 (32.9)	458 (67.1)	
	Unemploed	93 (18.3)	414 (81.7)	
	Worker and employee	263 (22.2)	922 (77.8)	
	Self-employed	345 (19.1)	1459 (80.9)	
	Student and soldier and unpaid work	79 (13.8)	492 (86.2)	

Abbreviation: WC, waist circumference. ^a^ *P* values are from chi-square test.

**Table 3:** WHR status according to socio-demographic characteristics in the study

**Characteristics**		**WHR status**		***P *** **value** ^a^
		**Above normal**	**normal**	
		**No. (%)**	**No. (%)**	
Age group (y)	20-29	802 (32)	1706 (68)	<0.001
	30-39	766 (45.4)	921 (54.6)	
	40-49	753 (55.8)	597 (44.2)	
	50-59	1397 (70.3)	591 (29.7)	
	≤60	1237 (76.5)	379 (23.5)	
Residency	Urban	3475 (54.3)	2923 (45.7)	0.6
	Rural	1480 (53.8)	1271 (46.2)	
Sex	Male	1980 (52.6)	1784 (47.4)	0.01
	Female	2975 (55.2)	2410 (44.8)	
Ethnicity	Baloch	108 (45.8)	128 (54.2)	<0.001
	Turk	1003 (50.8)	973 (49.2)	
	Turkoman	87 (66.4)	44 (33.6)	
	Sistani	33 (42.3)	45 (57.7)	
	Arab	119 (55.9)	94 (44.1)	
	Fars	2600 (56.5)	1998 (43.5)	
	Kurd	399 (57.5)	295 (42.5)	
	Gilak	165 (44.1)	209 (55.9)	
	Lor	385 (52.5)	349 (47.5)	
	Multiethnicity	18 (39.1)	28 (60.9)	
Education level	No education	1707 (71.3)	687 (28.7)	<0.001
	Primary/secondary/tertiary degree	1789 (52.8)	1601 (47.2)	
	High school degree	875 (43.4)	1141 (56.6)	
	University degree	582 (43.2)	764 (56.8)	
Family size	1	206 (66.5)	104 (33.5)	<0.001
	2-4	3289 (53.6)	2847 (46.4)	
	5 and above	1451 (54)	1234 (46)	
Occupation status	Housewife	2550 (58.1)	1837 (41.9)	<0.001
	Retried	481 (71)	196 (29)	
	Unemploed	203 (40.8)	294 (59.2)	
	Worker and employee	575 (49.1)	597 (50.9)	
	Self-employed	931 (52.4)	846 (47.6)	
	Student and soldier and unpaid work	189 (33.8)	370 (66.2)	

Abbreviation: WHR, waist-hip ratio. ^a^ *P* values are from chi-square test.

**Table 4:** Having the history of elevated BP, CVDs and T2DM by BMI, WC and WHR status in the study population

	**History of elevated BP**		**History of CVDs**		**History of T2DM**	
	**Yes**	**No**	**Yes**	**No**	**Yes**	**No**
	**No. (%)**	**No. (%)**	**No. (%)**	**No. (%)**	**No. (%)**	**No. (%)**
Underweight	20 (1.2)	188 (4)	10 (1.5)	377 (4.3)	13 (1.3)	374 (4.4)
Normal	348 (21)	1772 (38.2)	188 (27.3)	3392 (38.5)	187 (19.2)	3393 (39.8)
Overweight	648 (39.1)	1738 (37.4)	266 (38.6)	3144 (35.7)	400 (41.2)	3010 (35.3)
Obese	640 (38.6)	945 (20.4)	225 (32.7)	1896 (21.5)	372 (38.3)	1749 (20.5)
*P* value^a^	<0.001		<0.001		<0.001	
Above normal WC	1060 (65.2)	1682 (36.9)	390 (56.9)	3246 (37.7)	624 (66.2)	3012 (36.1)
Normal WC	567 (34.8)	2874 (63.1)	295 (43.1)	5363 (62.3)	319 (33.8)	5339 (63.9)
*P* value	<0.001		<0.001		<0.001	
Above normal WHR	1184 (74.3)	2352 (52.3)	482 (72.6)	4473 (52.7)	730 (79.3)	4225 (51.3)
Normal WHR	410 (25.7)	2147 (47.7)	182 (27.4)	4012 (47.3)	190 (20.7)	4004 (48.7)
*P* value	<0.001		<0.001		<0.001	

Abbreviations: BP, blood pressure; CVDs, cardiovascular diseases;T2DM, type 2 diabetes mellitus; BMI, body mass index; WC, waist circumference; WHR: waist-hip ratio. ^a^ *P* values are from chi-square test.
